# Functional somatic symptoms in Emergency Department frequent presenters

**DOI:** 10.1186/s12873-024-01030-w

**Published:** 2024-07-18

**Authors:** Vidula Garde, Katherine Thornton, Madelyn Pardon, Vinay Gangathimmaiah, Andrew J Mallett, Jaimi Greenslade, Kerrianne Watt

**Affiliations:** 1 Townsville Hospital and Health Service, Townsville, QLD Australia; 2 College of Public Health, Medical and Veterinary Science, James Cook University, Townsville, QLD Australia; 3 College of Medicine and Dentistry, James Cook University, Townsville, QLD Australia; 4 College of Healthcare Sciences, James Cook University, Townsville, QLD Australia; 5 Emergency and Trauma Centre, Royal Brisbane and Women’s Hospital, Brisbane Queensland, Australia; 6 Australian Centre for Health Services Innovation, Centre for Healthcare Transformation, Faculty of Health, Queensland University of Technology, Brisbane, QLD Australia; 7 Institute for Molecular Bioscience, The University of Queensland, Brisbane, QLD Australia

**Keywords:** Functional somatic symptoms, Medically unexplained symptoms, Frequent ED presenters, Psychological distress in ED, Somatization in ED, High value care in ED

## Abstract

**Background:**

Patients with Functional Somatic Symptoms (FSS) are frequently encountered within healthcare settings such as Emergency Departments (ED). There is limited research regarding characterisation and frequency of FSS within frequent presenters to ED and no previous Australian evidence. This study aims to fill this gap.

**Methods:**

A retrospective, single-centre study of frequent ED presenters over a 6-month period was undertaken. Patients with > 3 re-presentations/month were reviewed for the presence of FSS using Stephenson and Price’s (Stephenson DT, Price JR. Medically unexplained physical symptoms in emergency medicine. Emerg Med J. 2006;23(8):595.) categorisation of FSS. Patients were divided into three groups – FSS, possible FSS (pos-FSS) and non-FSS. The characteristics of these groups were compared using descriptive statistics (chi-square tests, Welch’s ANOVA). Person-time at risk during the 6-month study period was estimated for patients in each group and incidence of ED presentation for each group was then calculated. Psychological distress indicators for ED presenters with FSS, as noted by the treating clinician, were also analysed.

**Results:**

11% (71/638) of frequent ED presenters were categorised as having FSS and 72% (458/638) as having possible FSS (Pos-FSS). Mean ED presentations in the FSS group during the study period were significantly higher than in the non-FSS and Pos-FSS groups (*p* < 0.01). Anxiety was found to be the primary psychological distress indicator associated with ED presentations with FSS.

**Conclusion:**

We found that, amongst frequent ED presenters, patients with FSS presented significantly more frequently to ED than those without FSS. We propose revising the model of care for FSS in ED to promote appropriate referral to therapy services as a possible demand reduction strategy to improve patient care and efficiency in ED.

**Supplementary Information:**

The online version contains supplementary material available at 10.1186/s12873-024-01030-w.

## Background

Somatization defined as “a tendency to experience and communicate psychologic distress in the form of physical symptoms and seek medical help” [1] is ubiquitous within healthcare [2–4]. These disorders are variously referred to as Functional Somatic Symptoms (FSS), Somatoform Disorder, or Medically Unexplained Symptoms (MUS) [5]. Somatization disorders are common in the Emergency Department (ED) [6] and present both in adult [7]and paediatric [8, 9] populations. Somatization is associated with excess healthcare costs [10], iatrogenic harm and poor patient outcomes [11].

Frequent presenters to EDs tend to be complex and resource-intensive. [12–14] Individuals with somatization comprise a significant proportion of frequent presenters to ED [7, 15]. Prior research indicates that somatization is a significant contributor to health care costs in ED [10]. This cost is particularly significant in recent times due to stretched healthcare resources compounded by the COVID-19 pandemic [16, 17]. There is limited literature regarding the prevalence of somatization amongst ED presentations. Existing studies indicate various rates of FSS in frequent ED presentations ranging from 13.4% [7] to 28% [18]. These studies differ in their definition of frequent presentations (> 2 visits in 2 years vs. > 4 visits in six months) [7, 18] and their criteria for somatization. A recent UK study found an increase in ED presentations for medically unexplained symptoms despite an overall reduction in presentations during the COVID-19 pandemic [19]. There is no known Australian published literature about the prevalence of FSS in frequent ED presenters and the current study seeks to address this gap.

The study aimed to estimate the characteristics and prevalence of FSS in frequent ED presenters and to identify burden of disease attributable to FSS. The objectives of this study were to determine the following:

1) Proportion of frequent ED presenters that could be identified as FSS, possible FSS (Pos-FSS), and non-FSS.

2) Mean number of ED presentations for those categorised as FSS, Pos-FSS, and non-FSS.

and.

3) Differences in the characteristics of those categorised as FSS, Pos-FSS, and non-FSS.

## Methods

The study was conducted at the Emergency Department (ED) of Townsville University Hospital (TUH), Queensland, Australia. TUH is the tertiary referral centre for North Queensland servicing a catchment of ~ 700,000 people [20]. TUH had an annual ED census of 91,997 in 2020–2021 [21]. During the study period, there were 39,860 presentations amongst 27,064 patients.

### Patient selection and data abstraction

**Fig. 1:**
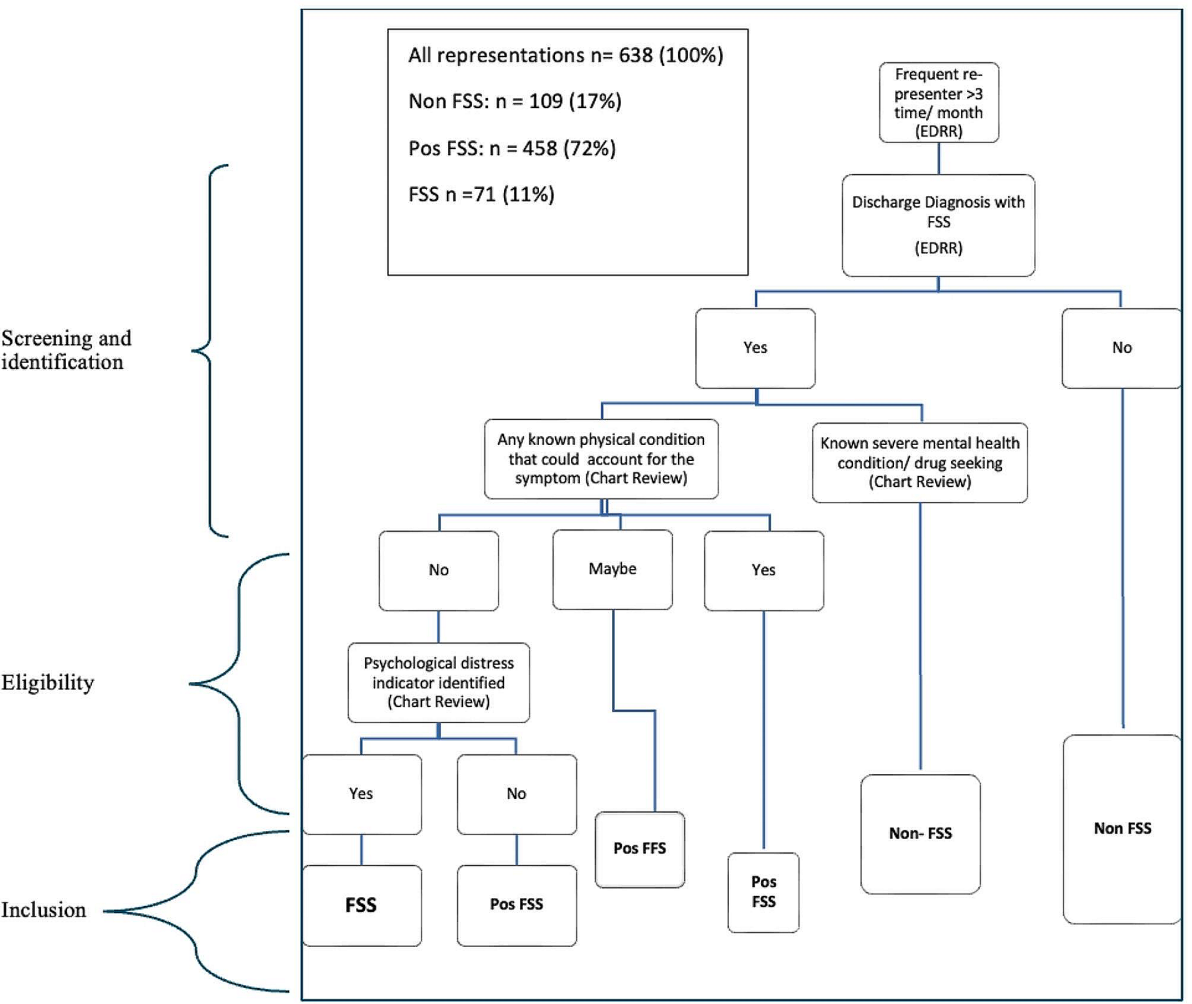
Screening, data abstraction and patient selection

This is a retrospective, single-centre study of patients who frequently presented to TUH ED between October 1st, 2016 - March 31st, 2017. Two investigators (VG1 and KT) independently determined patient eligibility for inclusion into FSS, Pos-FSS, or non-FSS groups. (Fig. 1), collated qualitative descriptions of stressors from patient charts and analysed data.

A consensus definition of frequent ED presenters is lacking although there have been attempts to operationalise the concept [22]. For this study, we defined a frequent ED presenter as someone who presents 3 or more times in a calendar month to ED. This definition was used for operational convenience since the organisation where this study took place produces an administrative report each month identifying these individuals. The report is called the ED recidivist report (EDRR) and contains details relating to patient’s name, number of presentations, arrival date and time for each presentation, and discharge diagnosis. Amongst our cohort of interest (people presenting 3 or more times in a month), patients were categorised into FSS, Pos FSS or non-FSS, based on the index episode (Fig. 1). The date of the first ED presentation during the first calendar month on which the patient was identified on EDRR was considered the index episode. If the index episode included any of the following diagnoses, the patient was categorised into FSS: : “non-cardiac chest pain, benign palpitations, nonspecific abdominal pain, non‐ulcer dyspepsia, physical symptoms of anxiety and depression, nonspecific symptoms (e.g. “funny turns”), symptoms with undiagnosed organic pathophysiology, no obvious pathology and non-specific presenting issues” Any patients whose diagnosis at index episode included a medical explanations for any symptoms, or who had a concurrent mental illness (e.g., Bipolar disorder, major depression, psychotic episode or schizophrenia, suicidal ideation or known drug seeking behaviour). were categorised as nonFSS. These diagnoses, taken verbatim from the EDRR, were guided by Stephenson and Price’s observations [6], in recognition of the fact that FSS present differently across different healthcare settings [23, 24].

We excluded those with mental health conditions and known addiction in the FSS category because, in their case, the presenting symptoms could have been influenced by other factors, e.g. drug seeking behaviours in case of individuals with known addictions; and delusions or hallucination in case of individuals with known mental health history. Only those whose symptoms were associated with psychological distress were categorised as FSS. Patients with an equivocal clinical diagnosis, such as cases where the index episode was FSS but for whom there was an established underlying pathology that could result in similar symptoms (e.g. non-cardiac chest pain in patients with established ischaemic heart disease), were classified as possible FSS (pos-FSS) group because the presentation for these patients may have been due to FSS. These patients were included in the study to enrich our understanding.

We recognise that this is somewhat arbitrary since the current DSM 5, classification would also consider those individuals who present with medically unexplained symptoms, despite suffering from a related condition, as falling within the somatic symptoms disorder category (“…the physical symptoms may or may not be associated with a diagnosed medical condition”). We also recognised that other researchers such as Theadom [18] et al. had considered these as MUS. However, we felt that since we were categorising based on file reviews, that we were better able to defend our categorisation if we found somatic symptoms not explained by other medical conditions and when these were associated with psychological distress. The classification of cases as FSS, Pos FSS or Non-FSS was carried out by two clinical psychologists (VG1 and KT).

Since there was no published Australian literature regarding operationalizing FSS amongst ED presentations at the time of study, previous EDRRs were used to familiarize the two psychologists regarding FSS using re-presentation reports and patient charts. This helped create shared understanding of FSS as described in the patient selection algorithm (Fig. 1). The psychologists undertook chart reviews of 144 frequent presenters identified through one of the earlier EDRRs to assess for inter-rater reliability. Presentations were independently rated as being FSS or Non-FSS and inter-rater reliability was calculated using Pearson’s co-efficient of correlation (*r* = 0.9).

### Data analyses

The data were analysed using Statistical Packages for Social Science (SPSS) version 26 and Microsoft Excel 10. Descriptive statistics were used to describe frequent ED presentations among those characterised as FSS, Pos-FSS and Non-FSS. Means with standard deviations were used for continuous variables and counts with proportions were used for categorical variables. Differences in age-group, gender and ethnicity between frequent presenters categorised as FSS, Pos-FSS and non-FSS were assessed using independent chi-square tests. Between group differences in mean ED presentations were assessed by Welch’s ANOVA (as assumption of homogeneity of variance was violated). Games-Howell post-hoc comparisons were conducted to assess between group differences.

### Person time at risk

An index episode was identified for each patient. To be included in the study, patients needed to be listed on EDRR which encompassed patients who attended ED, more than three times in one calendar month. The date of the first ED presentation during the first calendar month on which the patient was identified on EDRR was considered the index episode. The patient was considered to be “at-risk” of presenting to ED for the duration of the study period after the index event. Hence a patient who was included in the study from the first month of the 6-month study period contributed almost 6 months of person time at risk to the study, whereas a patient who was included in the study in the last month of the 6-month study period contributed only 1 month of person time at risk (the follow up periods were vastly different). Person—time at risk of ED presentation during the study period was calculated for each frequent presenter, and then separately for those categorised as FSS, Pos-FSS and Non-FSS. Subsequently, the incidence rate of ED presentation was calculated for the three groups (number of ED presentations/person- time at risk). Rates are presented per 100-person days. The relative risk of ED presentation (with 95%CI) was calculated for those categorised as FSS compared to non-FSS. Relative risks were also calculated for Pos-FSS compared with Non-FSS.

### Psychological distress indicators

Qualitative data regarding psychological distress indicators for those included in the study as FSS were descriptively analysed. Psychological distress indicators were defined as indicators for psychological distress noted by the treating ED clinician in the patient’s chart. For example, “the patient appeared highly anxious” or “patient reports a recent break up in relationship”. These were extracted directly from patient charts as recorded by the treating ED clinician (usually an ED nurse or a doctor). Researchers (VG1 and KT) then thematically grouped into the following categories: Anxious (Anxious mood and high anxiety); depressed, financial stress, living /residential stressor; psychosocial family/relationship issues; PTSD and psychosocial distress (including distressed and tearful). The last category was a category of exclusion when the distress indicator could not be classified in any other category. Categories were not mutually exclusive and patients could have more than one psychological distress indicator.

## Results

### Prevalence of FSS in frequent ED re-presenters

During the six-month study period there were a total of 39,860 ED presentations amongst 27,064 patients. This included 638 unique frequent ED presenters, with 71 (11%) categorised as having FSS, 109 (17%) as Non-FSS, and 458 (72%) as Pos-FSS (Fig. 1).

### Age distribution

Sample characteristics by FSS category are shown in Tables 1 and 2. Those aged 50-59-years accounted for the highest proportion of frequent presenters categorised as FSS followed by those 20 to 29 and 30–39 years. There is also a peak of FSS frequent presenters relative to Pos FSS and Non FSS, between the ages of 70–79 years.

**Table 1:** Comparison between FSS subgroups - patient characteristics

Patient Characteristics	FSS (*n* = 71)	Pos FSS (*n* = 458)	Non-FSS (*n* = 109)	Total *N* = 638
Gender				
Male Female	30 (42.3%) 41 (57.7%)	232 (50.7%) 226 (42.3%)	50 (45.9%) 59 (54.1%)	312 (48.9%) 326 (51.1%)
Ethnicity**				
Aboriginal / Torres Strait Islander	7 (9.9%)	140 (30.6%)	31 (28.4%)	178 (27.9%)
All other	64 (90.1%)	317 (69.4%)	78 (71.6%)	459 (72.1%)
Age-group				
20-29yrs 30-39yrs 40-49yrs 50-59yrs 60-69yrs 70-79yrs 80 + yrs	12 (16.9%) 12 (16.9%) 9 (12.7%) 15 (21.1%) 7 (9.9%) 10 (14.1%) 6 (8.5%)	82 (21.1%) 83 (21.3%) 82 (21.1%) 52 (13.4%) 30 (7.7%) 33 (8.5%) 27 (6.9%)	20 (18.3%) 23 (21.1%) 27 (24.8%) 13 (11.9%) 11 (10.1%) 7 (6.4%) 8 (7.3%)	

** The difference is significant at the 0.01 level

**Table 2:** Comparison between FSS subgroups – Mean ED presentations

	Mean (SD)	95%CI	Min ED presentations	Max ED presentations
FSS (*n* = 71)	6.35 (6.007)	4.93–7.77	3	38
Non-FSS (*n* = 109)**	4.50 (1.809)	4.16–4.85	3	12
Pos-FSS (*n* = 458)**	4.05 (1.823)	3.84–4.15	3	26

*Notes* Post-hoc (Games Howell) comparisons indicated that the mean difference between FSS and non-FSS, and FSS and Pos-FSS were significant (*p* < 0.001, respectively). Games-Howell post-hoc comparisons were conducted because the assumption of homogeneity of variance was violated

Non-parametric data are shown for interest. FSS: Median = 4.0 (IQR = 3); Pos FSS: Median = 4.0, (IQR = 1); non FSS: Median, 4.00 (IQR = 2.0). A non-parametric (Kruskal-Wallis) test yielded the same result as reported for ANOVA (X^2^ = 15.58; df = 2, *p* < 0.001). However, the post-hoc pairwise comparisons were different. While there were significant differences between FSS and pos FSS (*p* < 0.01), and non FSS and Pos FSS (*p* < 0.01), there was no significant difference between Non FSS and FSS (*p* > 0.05)

### Gender and ethnicity

There was no association between patient sex and the presence of FSS (*X*^*2*^ *= 2.22 df 2p = 0.33*) (*p* > 0.05); (Table 1). FSS categorization varied by ethnicity (Table 1) (X^2^ = 13.19; df = 2; *p* < 0.01). Despite constituting 27.9% of frequent presenters, persons identifying as Aboriginal/Torres Strait /South Sea Islander accounted for only 9.9% of FSS presentations, compared with 30.6% of Pos FSS and 28.4% of Non-FSS (Table 1).

### FSS amongst frequent ED presenters

Frequency of ED presentation differed by whether patients were categorised as FSS, Pos-FSS and non-FSS (F = 8.32; df = 2, 130.38, *p* < 0.001). (Games-Howell) post-hoc comparisons showed that mean ED presentations during the 6-month study period (Table 2) were significantly higher among frequent ED presenters categorised as FSS (X = 6.35 presentations; SD = ± 6.007) than either Pos-FSS (4.05 presentations; SD = ± 1.823; *p* < 0.001)) or non-FSS (4.50 presentations; SD = ± *1.809;p < 0.001)* Additionally, ED presentations were higher among frequent presenters categorised as Non-FSS compared with Pos-FSS (*p* < 0.05).

### Contributions of FSS categories to burden in ED re-presentations

To assess the proportionate extent of the impact of FSS on the number of frequent re-presentations, we compared the total ED presentations for each FSS category The 638 frequent presenters yielded a total of 2808 ED presentations during the 6-month study period. The 11% of frequent ED presenters who were categorised as FSS accounted for 16% of the total ED presentations made by frequent presenters. Comparatively, the 17% of frequent ED presenters who were categorised as Non-FSS accounted for 19% of total presentations made by frequent presenters.

The incidence of ED presentations was calculated for each group. Among frequent presenters categorized as FSS, the incidence of ED presentations during the study period was 4.18 per 100 person days (95% CI: 3.76–4.61). This was 1.29 times higher (95% CI: 1.16–1.43) than frequent presenters categorized as Non-FSS (IR: 3.25per 100 person-days; 95% CI: 2.93–3.57), and 1.47 times higher (95% CI: 1.32–1.63) than frequent presenters categorized as Pos-FSS (IR: 2.85 per 100 person days; 95% CI: 2.71–2.99). The proportion of ED presentations among those categorized as FSS attributable to FSS was 30.14% (Attributable Fraction). Among this group of frequent presenters (FSS, Pos FSS, and Non-FSS), 4.6% of all ED presentations during the study period were attributable to FSS (population attributable fraction).

### Psychological distress amongst ED frequent presenters with FSS

Anxiety was the main psychological distress indicator associated with frequent ED presentation for FSS (64.7%;). Other indicators included psycho-social, family and relationship stressors (25.3%), depression (18%) and stressors associated with place of residence (~ 10%).

Based on the discharge diagnoses, non-specific chest pain diagnoses and palpitations were the most frequent presentations (49.5%) followed by non-specific abdominal pain diagnoses (35.9%). The two together accounted for 85.4% of all discharge diagnoses for FSS patients. Additionally for 1 out of 5 presentations, the frequent presenter did not wait for diagnosis.

## Discussion

Despite the ubiquitousness of FSS in healthcare systems, there is limited research regarding the frequent ED presenters with FSS in Australian EDs. We analysed frequent ED presenters (> 3 ED presentations/month) for FSS over 6 months and found that 11% of frequent ED presenters presented for FSS, accounting for a disproportionately large number of ED presentations (16%). We further found that individuals with FSS presented more frequently than individuals with either possible FSS (Pos-FSS) or those without FSS (Non-FSS).

Our findings suggest that individuals with FSS have higher utilisation of healthcare services including a higher incidence of ED visits than those without FSS. This finding is broadly consistent with existing literature [7, 10, 18]. We have found lower proportion of FSS in our study compared to the studies of Alsma et al. [7] and Theadom et al. [18]. This is likely due to different eligibility criteria for study inclusion. Alsma et al. [7] included all ED patient presentations and Theadom et al. [18] included ED patients with > 4 presentations over 6 months. We included only ED patients with ≥ 3 presentations in a calendar month. By our definition those patients who would have presented to ED twice in every calendar month during our study period would therefore not have been included as FSS in our sample, whilst they would have been included in the other studies.

Secondly, our results could also be attributed to the stringency of our selection criteria. Alsma et al. [7] included patients with *“symptoms without an adequate explanation despite adequate assessment of history and physical examination, diagnostic testing in the ED or during follow-up.*” In contrast, our criteria did not merely require that the symptom be medically unexplained, but that there also be some evidence of psychological distress. Additionally, our inclusion criteria required agreement for inclusion by two clinical psychologists and study team members unlike Theadom et al. [18] who used a single researcher (a clinical psychologist) for categorisation. Furthermore, Theadom et al. [18] used a binary approach to categorisation where individuals with symptoms not attributable to an organic cause were categorised as FSS or no FSS. This would likely have led to the categorisation of patients with medically unexplainable chest pains and a history of previous heart attack as FSS whilst our study would have categorized such patients as possible FSS (Pos-FSS). Our study also applied a rigorous diagnostic algorithm and a separate category of Pos-FSS for similar conditions. We also identified a similar proportion (57.7% vs. 57.6%) of female patients amongst frequent ED presenters with FSS as Alsma et al. [7].

Anxiety was the leading concomitant psychological distress indicator in our study, as with Theadom et al’s [18] study. Alsma et al. [7] et al. found depression and panic disorder in almost a third of their sample and anxiety disorder in a fifth of their sample [11]. Since the study relies on the notes made by the consulting clinician in ED, it is possible that the information pertaining to psychological distress could be affected by the clinician’s personal orientation, experience and rapport with the patient. There could have been other psychological comorbidities which have not adequately been captured or that the ones captured might not fully represent the individual’s subjective state.

Our findings could have implications for service needs of FSS amongst ED presentations and for service redesign to meet current clinical needs. The standard treatment offered for FSS in ED settings involves providing advice regarding positive health behaviours, reassuring such patients regarding their health, encouraging them to contact their GP and, return to the ED if symptoms persist. Since anxiety appears to be the most pressing concomitant psychological concern, it is likely that any reassurance that is offered in ED regarding the apparent absence of a physical disorder will have only a temporary effect. This presence of high anxiety is likely to result in individuals repeatedly presenting either to seek reassurance or as a response to resurgence in anxiety over time. An effective framework for reducing the risk of re-presentation is therefore likely to require improved anxiety management and provision of comprehensive care, rather than further investigations, in keeping with treatment guidelines [3, 5]. Possibly, the instituting of anxiety management strategies as an adjunctive measure whilst the patient is in ED could be of assistance. Additionally, the ED clinician could also request the GP to assess for ongoing anxiety or other psychological difficulties and refer as appropriate. Where individualised care plans exist, ongoing psychological support could be explored as an addition to standard care. We would like to suggest that all individuals who tend to re-present should be targeted relatively early in their journey and referred to psychological services for assessment and management of any comorbid psychological condition. We would also like to suggest that this should be done relatively sensitively, so that he patient, upon discharge from ED does not move forward with the impression that their pain and suffering has been invalidated by a non-compassionate system.

### Implications

The most important clinical implication of our study is to guide the provision of high-value care in ED. In keeping with earlier researchers in the field [3, 5, 7, 18] we concur that early identification of patients experiencing FSS could lead to different and potentially more appropriate treatment. This will likely improve the quality of care that these patients receive in addition to patient experience and outcomes. It may potentially also have beneficial impacts upon ED workload [7] by reducing ED presentations and reducing overall health service utilisation and costs. This is particularly relevant given the emerging literature identifying an increase in FSS presentations to EDs after the commencement of the COVID-19 pandemic [25].

Our findings suggest that frequent ED presenters with FSS contribute a disproportionately high number of service-episodes and are at an increased risk of re-presentation. We therefore suggest that individuals who frequently re-present to ED should be screened for FSS and referred to appropriate services. This could be particularly relevant since our largest group is the Pos-FSS group.

There are no Australian data regarding the overall cost of somatization amongst ED presentations, although international literature indicates that it could be very high [10, 26]. It would be of particular interest to identify the real-world direct and indirect costs of somatization to assess the feasibility and potential economic benefit of creating alternate clinical pathways and service redesign.

### Limitations

These are preliminary findings of a single-centre study over a limited time with inherent limited generalizability. There is a need to replicate our findings across additional centres to develop a more comprehensive understanding of FSS and its identification. This will assist in effectively identifying patients and the true extent of the problem. Further, our study used very stringent criteria for FSS. Had we applied less strict criteria then we might have found a higher prevalence in keeping with previous research.

Our sampling included only those individuals who presented three or more times in any calendar month and thus likely underestimates the true extent of FSS and FSS episodes in ED – both cross-sectionally (< 3 episodes/month not counted) and longitudinally (any FSS presentation after or before 6 months not counted). We acknowledge that the study could be improved if the index presentation was considered along with any presentation in the three months prior, for potential inclusion/exclusion criteria. A strength of the study is that there is only one public hospital in the area, and so the sample is representative of those who regularly seek urgent medical attention in the region. Our FSS sample does not include those who have a physical condition that might account for the symptoms or those who have a mental health condition. This limits the generalizability of our findings. We have also used information regarding psychological distress indicators from electronic medical records. We acknowledge that these could be lacking in symptoms documentation and could be subjected to observer bias. Another limitation of our study is that we have classified mental health presentations as Non FSS rather than as a separate category. This has resulted in categorisation of mental health presentations in the same category as physical condition such as physical trauma. Since there is literature supporting the fact that mental health conditions present relatively more frequently to ED [27], this categorisation could potentially have affected the total presentations for Non FSS group.

As a preliminary study, ours was designed to assess the burden of frequent ED presentations conferred by patients with FSS with our findings suggesting that further research is warranted. It would be of interest if our study could be replicated by other EDs and our algorithm standardized. This could enable an alternative care pathway to be created for appropriate diversion of individuals presenting to ED with somatization. Future areas for more detailed exploration also include identifying the actual burden conferred by such patients in ED settings (time spent in ED, procedures and tests performed, etc.), across different clinical settings (e.g., prehospital, primary healthcare), and what strategies might successfully divert their presentations to appropriate services.

## Conclusions

We found that 11% of frequent ED presenters were categorised as FSS, contributing to 16% of ED presentations over a six-month period. The mean number of ED presentations for those identified to have FSS was significantly higher than for other patients. Among the frequent presenters, the presence of FSS increased the risk of presentations to ED. Anxiety was the most frequent psychological distress indicator and was present in two-thirds of those presenting with FSS to ED. Appropriate and early identification and treatment for patients with FSS presenting to EDs could provide more appropriate patient care and, over a period, potentially serve to reduce ED presentations. These findings have ongoing implications for delivering high-value emergency clinical care.

### Electronic supplementary material

Below is the link to the electronic supplementary material.

Supplementary Material 1

## Data Availability

The datasets used and/or analysed during the current study are available from the corresponding author on reasonable request.
