# Supplementary material for: Functional somatic symptoms in Emergency Department frequent presenters
Source: BMC Emerg Med. 2024 Jul 18;24:122. doi: 10.1186/s12873-024-01030-w (PMC11256397; doi:10.1186/s12873-024-01030-w)
Supplement: Supplementary file 1 — Supplementary Material 1 [file 12873_2024_1030_MOESM1_ESM.docx]

**Supplementary Material**

Supplementary Figure 1. Psychological distress indicators in FSS in ED frequent re-presenters

*NB. Overall percentages do not add to 100 because some individuals had more than one psychological distress indicator listed in their charts.*
